# Supplementary material for: Patterns of Intron Gain and Loss in Fungi
Source: PLoS Biol. 2004 Nov 30;2(12):e422. doi: 10.1371/journal.pbio.0020422 (PMC532390; doi:10.1371/journal.pbio.0020422)
Supplement: Table S1 — Also available at http://genes.mit.edu/NielsenEtAl/. (4.3 MB ZIP). [file pbio.0020422.st001.zip › NielsenEtAl/html/86.html]

AN3190.1.NCU01879.1.MG10578.1.FG02794.1


```
 CLUSTAL W (1.82) Multiple Sequence Alignments - Introns Inserted


Sequence 1: MG10578.1	379 aa
Sequence 2: FG02794.1	355 aa
Sequence 3: NCU01879.1	391 aa
Sequence 4: AN3190.1	399 aa
Alignment Length: 431 aa
Number Identitical Residues: 100 aa
Alignment Score (without introns) 5877


MG10578.1 	MLSFWPWR0GDNSSTASFEKALSSLSAKINATQAQLDRTRSTARRVQVLWSLYLSFAYLI
NCU01879.1	MVSLWPWK0KEDSSPASFEKALSALSGRITVTQTRLDNVRSTARRVKGLATLYLGFAYLV
FG02794.1 	MVAFWPWG0GESSDTASFEKTLSTLSTKINDAQASLDNFRASSRRARVIWTLYLSFAYLV
AN3190.1  	MVSLWPFK0GEDTSPAGFEKALSALSEKITQTTTRLDLHRQHARRFKALWTLYTTFIYLL
          	*:::**:   :.:..*.***:**:** :*. : : **  *  :** : : :**  * **:

MG10578.1 	YAIVLVLVVGWTNLGPYEWSGVAGGPVL~IYVTRTALSAFFNYRIDSLSTRLKGQKEERA
NCU01879.1	YAIVALLVIGWKNMGPQEWTGMAGGPVI~IYLVRTLSTMFFDYRINGLNAKLEYLQTERA
FG02794.1 	YAIVLLLVVGYDNLGAFEWGGLTGGPLL2-------------FRIETLSARLREHQQERA
AN3190.1  	YSIILALVLGWENWGVKEYAAVLGGPVI2IYLVRAGGTMFFEYRINHTQRYLDSLQKQRD
          	*:*:  **:*: * *  *: .: ***::      :  :   .:**:  .  *   : :* 

MG10578.1 	KTVQKLKEATKYDSTLELLEKYGGEPKRPRGKKGSIGGEDGGDKQGEGSKQPTRPGSANP
NCU01879.1	KTIQKLKDATRYDSTLELLEKYGGPENKIRRRASKKGGDDDNDED--GAQRSKKSQGRHA
FG02794.1 	KTIQKLKDATKYDSTMELIEKYGGADNKSKKKDG----EQNADKT-AGKQQHTAPGQGPP
AN3190.1  	ETIEKLKVATKYNSTQQLLEKYAGDSPKPSRSKGGDHKPKPENKR-KPSGQQQQPPQPQF
          	:*::*** **:*:** :*:***.*   :     .     .  ::      :   .     

MG10578.1 	GRTGIAPPPTANIQRR-----SAPSTPQPPR------AAQFPPPREVSAEFAPNAFGPGG
NCU01879.1	NRIHIAPPPTANIQRPGAVGPNMPQPGAPPRGQQLAPSPMPPHGPEPSAEFAPNAFETN-
FG02794.1 	GRTRMPPPPTANIQRA-----HSPVPG--------------PTLIEPGAEFAPNAEWSG-
AN3190.1  	HRTGLPPPPTANIRRP-----------------------VPPPPEQPPSPYPSQQYQQPR
          	 *  :.*******:*                          *   :  : :..:      

MG10578.1 	PPDSPATMGSAQYAPLPP---------SAGSEPHWYDRIMDLLLGDDEMAAKNRIVLICA
NCU01879.1	PRASPAPP-HAQYPLAAAA-------MAAPTESHWYDRILDTLIGEDETAAKNRIVLICK
FG02794.1 	PPPPPSAPGTPQPPPPPPPQRSYSSYSTAASETHWYDRIFDVLLGEDETAPKNRIVLICQ
AN3190.1  	PNMPPPGLDEPGFAPNAFP------TQQYIEQSRWYDRLLDVLLGEDETQPKNRIVLICA
          	*  .*.  . .  .  . .            :.:****::* *:*:**  .******** 

MG10578.1 	HCRLVNGQAPPGVKTLADVGRWRCMGCRAENGEDKVDECKRLVKEVLAARSG------SD
NCU01879.1	KCRLVNGQAPPGTKSLSEIGKWKCMSCGAVNGE--MDEGKRIVREVIGGGRSEESLVHSQ
FG02794.1 	SCRVVNGQAPPGTKTLSELGQWRCMSCGASNGE--LDEGRRIMNEVLDAAKS------AE
AN3190.1  	ACRLVNGQAPPGVKSLEEIGRWRCGGCGAWNGE--DSAARKVLADIKAAAGPVSEPSDGE
          	 **:********.*:* ::*:*:* .* * ***   .  :::: ::  .    ..   .:

MG10578.1 	AGDQVEASAEFKSD-SDGDSSDIVQIDREM--------------------AEAEESKGAT
NCU01879.1	SGDDATSLGSFGEEFADEELVDRSEAAE----------------------SDKVQERPEK
FG02794.1 	IGGGSAHEEFARED-SEPPMVDVSTPN------------------------DGPASNTRK
AN3190.1  	AETEHRFSTSTSDGASDGVIVTKEDDEQERNEVEVESQSGSGSGAETPAEDDEPASKIRT
          	            .  ::           . .. . .:.:.:.:.:.:.:. :   ..  .

MG10578.1 	KRKGKGKK-----
NCU01879.1	RLKTEGSK-----
FG02794.1 	RKGGNGTK-----
AN3190.1  	RSQNVTKRRARKG
          	:     .: : ..
```
